# Transcriptome and Resequencing Analyses Provide Insight into Differences in Organic Acid Accumulation in Two Pear Varieties

**DOI:** 10.3390/ijms22179622

**Published:** 2021-09-06

**Authors:** Qionghou Li, Xin Qiao, Luting Jia, Yuxin Zhang, Shaoling Zhang

**Affiliations:** State Key Laboratory of Crop Genetics and Germplasm Enhancement, Centre of Pear Engineering Technology Research, Nanjing Agricultural University, Nanjing 210095, China; 2019204004@njau.edu.cn (Q.L.); qiaoxin@njau.edu.cn (X.Q.); 2017204005@njau.edu.cn (L.J.); 2018104043@njau.edu.cn (Y.Z.)

**Keywords:** organic acid metabolism, WGCNA, regulatory network, domestication

## Abstract

Fruit acidity is one of the main determinants of fruit flavor and a target trait in fruit breeding. However, the genomic mechanisms governing acidity variation among different pear varieties remain poorly understood. In this study, two pear varieties with contrasting organic acid levels, ‘Dangshansuli’ (low-acidity) and ‘Amute’ (high-acidity), were selected, and a combination of transcriptome and population genomics analyses were applied to characterize their patterns of gene expression and genetic variation. Based on RNA-seq data analysis, differentially expressed genes (DEGs) involved in organic acid metabolism and accumulation were identified. Weighted correlation network analysis (WGCNA) revealed that nine candidate TCA (tricarboxylic acid)-related DEGs and three acid transporter-related DEGs were located in three key modules. The regulatory networks of the above candidate genes were also predicted. By integrating pear resequencing data, two domestication-related genes were found to be upregulated in ‘Amute’, and this trend was further validated for other pear varieties with high levels of organic acid, suggesting distinct selective sweeps during pear dissemination and domestication. Collectively, this study provides insight into organic acid differences related to expression divergence and domestication in two pear varieties, pinpointing several candidate genes for the genetic manipulation of acidity in pears.

## 1. Introduction

Fruit acidity is an essential component of the organoleptic quality of fruits. In general, malic acid (MA) is the predominant organic acid in pear fruit, followed by citric acid (CA) and oxalic acid (OA) [1]. In fruits, the biosynthesis of organic acids and other secondary metabolites occurs within their cells [2,3]. The tricarboxylic acid (TCA) cycle and glycolysis supply the components necessary for the carbon skeletons of all synthesized metabolites in fruit. Several enzymes are involved in the TCA cycle, including phosphoenolpyruvate carboxylase (PEPC), malate dehydrogenase (MDH), NADP-malic enzyme (NADP-ME), aconitase (ACO), NAD-dependent isocitrate dehydrogenase (NAD-IDH), and succinate dehydrogenase (SDH), among others. 

Investigating gene related to fruit acidity is important not only for pear breeding, but also for the breeding of many fruit trees. In apple (*Malus domestica*), three genes encoding NAD-dependent malate dehydrogenase (cyMDH), phosphoenolpyruvate carboxylase (PEPC), and cytosolic NADP–dependent malic enzyme (ME) were first cloned and their roles in malate accumulation were verified [4,5]. In sour lemon (*Citrus limon*), a gene encoding NADP-IDH, and expressed in the cytoplasm and mitochondria can synergistically regulate the citric acid contents of fruit cells [6], and a cascade of *CitAco3*–*CitIDH1*–Citrus glutamine synthetase 2 (*CitGS2*) may be associated with citric acid degradation [7]. Additionally, the proton pump is also important in organic acid accumulation. In *Arabidopsis thaliana*, one tonoplast dicarboxylate transporter gene (*AttDT*) and two aluminum-activated malate transporter (*AtALMT*) genes mediate malate transport [8,9,10]. The *PH*, *ALMT*, *ALMT-like*, *PH-like*, and V-type ATPase (*V-ATPase)* genes contribute to organic acid accumulation in orange (*Citrus sinensis*), apple and pear [11,12,13]. Bulked segregant analysis (BSA) and quantitative trait locus (QTL) analyses were also used to map the genes associated with fruit acidity. In apple, three hierarchical epistatic genes (small auxin up-regulated RNA 37 (*MdSAUR37)*, aphosphatase2C (*MdPP2CH*), and *MdALMTII*) were identified using a BSA, which can precisely determine the malate content in apple fruit malate content [14]. Through QTL mapping for fruit acidity, some candidate genes that encode organic acid metabolism- and proton transport–related genes were identified in *Cucumis melo* [15]. Recently, two QTLs for fruit acidity were identified, malic acid (*Ma*) and *Ma3*, were identified, providing specific DNA-based information for apple cultivar improvement [16]. Compared with BSA or QTL mapping, transcriptome sequencing can also provide candidate gene information for the identification of target traits at a lower cost. With the rapid development of clustering algorithms, RNA-seq data-based gene co-expression network analysis has become an effective way to investigate agronomic trait-related genes and their regulatory networks [17,18,19,20].

In their study on European pear, Nham et al. [21] used transcriptome data to identify several candidate genes and transcription factors (TFs) in four stages of maturity. Busatto et al. subsequently identified candidate genes by comparing the transcriptome of on-tree maturation and postharvest ripening in fruit of cv. ‘Abate Fetel’ [22]. By comparing fruit RNA-seq data from different storage times, 13 genes were found related to ethylene biosynthesis and signal transduction were identified in Asian pear, along with 2457 transcription factors [23]. The same approach was used to identify genes related to the core browning mechanism during pear storage, as well as the genes involved in nitrogen metabolism pathways [24,25]. These studies compared the same pear varieties at different developmental stages or under different treatment conditions. By applying clustering methods, Zhang et al. [26] identified several candidate genes involved in stone cells, sugar, acidity, and hormones levels across seven successive developmental stages in five different cultivated pears species.

Plant domestication created considerable differences between wild species and their modern variants, modifying traits such as seed dispersal, dormancy, flowering time, and fruit size [27]. Domestication can also alter gene expression levels between wild species and modern varieties. In common bean (*Phaseolus vulgaris*), nucleotide diversity has been reduced along with the expression of certain genes [27]. In maize (*Zea mays*), the expression levels of certain genes were altered during domestication, such as teosinte branched 1 (*tb1*), grassy tillers 1 (*gt1*), tassels replace upper ears 1 (*tru1*), and teosinte glume architecture 1 (*tga1*) [28]. Comparison of wild and domesticated tomatoes resulted in the detection of expression level changes in thousands of genes, with many of these changes resulting from different selection pressures [27,29]. The extent of these changes can be explained by domestication not only affecting individual genes in cultivated species, but also their regulatory networks [29,30]. Recently, a large-scale transcriptomic analysis in *P. pyrifolia* indicated that the ge58ne expression diversity can be associated with domestication of pear [20]. In that study, 113 pear (*Pyrus* spp.) accessions were sequenced, and many important traits-related genes were detected in selected regions, such as gene impacting sugar content, stone cells, and fruit size. However, few organic acid-related genes were detected in this study. The pear data were released in the NCBI SRA database [31] which provided resources that re-analyzed the phylogenetics and selective sweep regions for some specific pear populations. Using resequencing data, the analysis can be extended to include *P. pyrifolia*, *P. communis*, *P. bretschneideri,* and *P. sinkiangensis*.

In this study, we selected two *Pyrus* species (*P. bretschneideri* and *P. sinkiangensis*) with similar product maturity stage and contrasting fruit acidity, with the ‘Dangshansuli’ (DSHS, *P. bretschneideri* cv. Dangshansuli) variety having low levels of organic acids and the ‘Amute’ (AMT, *P. sinkiangensis* cv. Amute) variety having high levels of organic acids [32]. We then carried out transcriptome analysis and analyses of physiological characteristics analysis for both varieties. The MA and OA levels of ‘Amute’ (AMT) were found to be significantly higher than ‘Dangshansuli’ (DSHS). Expression profiles and WGCNA analysis showed that the gene expression pattern was quite different between the two varieties. Based on the combination of WGCNA and selective sweeps analyses, two domestication genes that may contribute to organic acid accumulation were identified.

## 2. Results

### 2.1. Quantitation of Organic Acid Contents in ‘Dangshansuli’ and ‘Amute’ Pear

**Figure 1:**
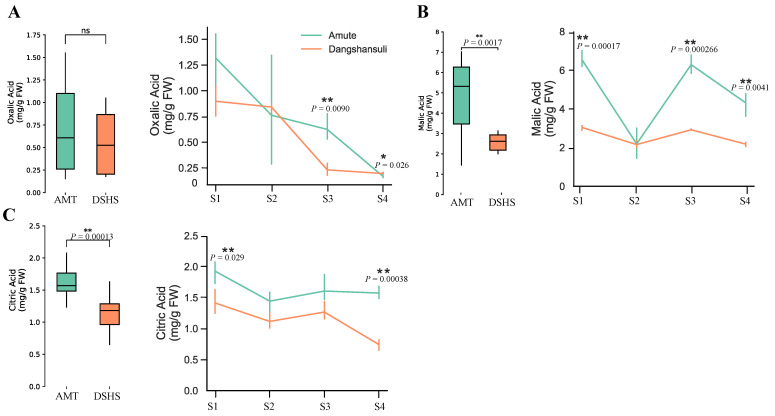
The contents of three organic acids in four fruit developmental stages of AMT and DSHS pears. (**A**) The contents of oxalic acid content. (**B**) The contents of malic acid content. (**C**) The contents of citric acid. The left boxplot displays overall organic acid levels in AMT and DSHS, while the right line plot represents the organic acid levels in four developmental stages in both AMT and DSHS. Blue and orange lines represent AMT and DSHS pears, respectively. S1/S2/S3/S4 on the X axis represents 15/45/90/120 days after full bloom; mg/g FW on the Y axis represents mg/g fresh weight (FW); Student’s *t*-test * *p*-value < 0.05; ** *p*-value < 0.01; ns: no significant.

We first compared the total level of three organic acid. MA was found to be a major component in both AMT and DSHS, followed by CA and OA. All three types of organic acids were higher in AMT than in DSHS (Figure 1, Table 1). Specifically, both MA and CA levels in AMT were shown to be significantly higher than in DSHS (Figure 1B,C, Table 1). OA showed no significant differences between these two varieties (Figure 1A). When scanning the OA levels in four successive developmental stages, we found that AMT had significantly higher levels during S3 and S4 than DSHS. Furthermore, MA was shown to be significantly higher than in DSHS during S1, S3, and S4. CA levels in AMT were significantly higher in DSHS during all four stages (S1-S4). These results suggest that the major organic acid in both AMT and DSHS was MA, and that MA and CA were significantly higher in AMT than in DSHS during most developmental stages.

### 2.2. Phylogenetic Analysis and Expression Landscapes between Two Varieties

**Figure 2:**
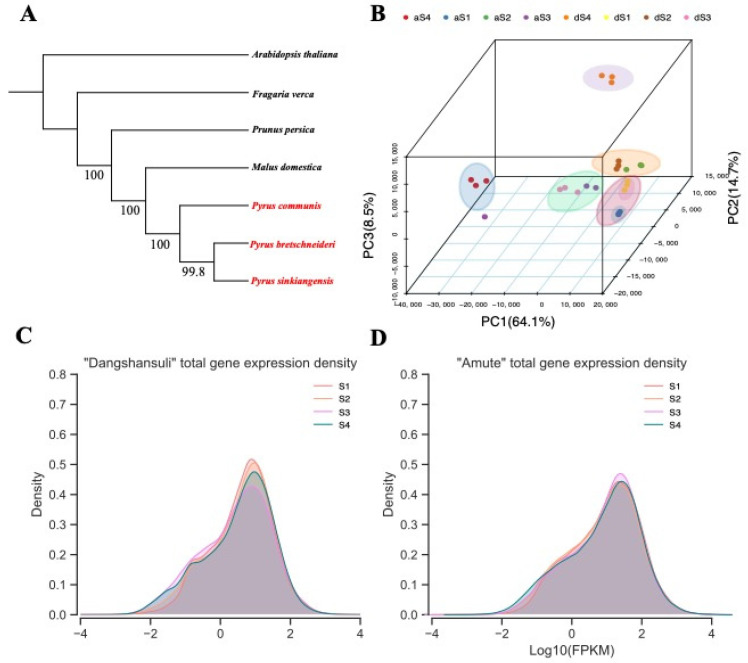
Phylogenetic tree, PCA, and overall expression distributions in two pear varieties. (**A**) Phylogenetic tree generated by ASTRAL on the basis of all single-copy genes. Branch labels are bootstrap values. ‘Bartlett’ (*P. communis*), ‘Dangshansuli’ (*P. bretschneideri*) and ‘Amute’ (*P. sinkiangensis*) were using RNA-seq data from this study and others were using genome data obtained from GDR. (**B**) PCA of 24 samples. In aS1–aS4, ‘a’ represents AMT and S1–S4 represents 15–120 DAFB; that is, aS1–aS4 represents AMT at 15–120 DAFB. The dS1–dS4 represents DSHS at 15–120 DAFB. (**B**) Kernel plot of overall expression density of DSHS. The Y-axis represents gene density, and the X-axis represents Log10(FPKM). (**C**) Kernel plot of the overall expression density in DSHS; S1-S4 represents 15-120 DAFB in DSHS. (**D**) Kernel plot of the overall expression density in AMT; S1–S4 represents 15–120 DAFB in AMT.

There is considerable evidence to suggest that Asian and European pears were experienced independent domestication processes [31,33,34]. However, *P. sinkiangensis* was inferred to originate from the inter–species hybridization of *P. communis* (European pears) and *P. bretschneideri* or *P. pyrifolia* (Asian pear) [31]. Both *P. communis* and *P. bretschneideri* have high-quality reference genomes, whereas the reference genome for AMT (*P. sinkiangensis*) was not available. Thus, we assembled the RNA-seq data of ‘Amute’ (*P. sinkiangensis*), ‘Dangshansuli’ (*P. bretschneideri*) and ‘Bartlett’ (*P. communis*) to explore the phylogenetic relationship. Other Rosaceae species which have extremely closed phylogenetic relationships with *Pyrus* spp. were included as outgroups in construction of a phylogenetic tree, including apple (*Malus domestica*), peach (*Pruns persica*), and woodland strawberry (*Fragaria vesca*). In addition, *Arabidopsis thaliana* was selected as an outgroup of Rosaceae. Both coalescence and concatenation trees supported that the phylogenetic distance between AMT and DSHS was closer than between AMT and ‘Bartlett’ (Figure 2A). When using *P. bretschneideri* genome as reference, the average mapping rate of RNA-seq data in AMT was 73.58% and in DSHS was 77.81% (Supplementary Table S1) which indicates that it is satisfactory for further analysis. Therefore, the genome of *P. bretschneideri* was selected as a reference for AMT.

The gene expression profiles were decoded and analyzed from corresponding RNA-seq data. In total, 198 Gb of RNA-seq data for eight samples with three biological replicates (24 RNA-seq libraries) were obtained, representing 12–20× coverage of the pear genome (Supplementary Table S1). The Pearson’s correlation coefficients (PCCs) between any two replicates of the same sample were between 0.8 and 1, except for the ‘A-90-2’ sample (Supplementary Figure S1). Interestingly, the median expression level of all the genes in AMT gradually declined during development, while in DSHS the expression level decreased in the first three stages of development, and then increased in the final stage (Supplementary Figure S2).

Principal component analysis (PCA) was performed on all 24 samples (Figure 2B). We can clearly observe that in the stages of S1 (15 DAFB) to S3 (90 DAFB), samples from the two varieties were clustered together, except for an outlier sample (‘A-90-2’). Samples from S4 were obviously divergent in the PCA plot. These results indicated that AMT and DSHS have similar expression profiles in the early stages of development (S1–S3; 15 DAFB-90 DAFB). Furthermore, the distribution of whole-genome gene expression (FPKMs) values revealed that AMT has a higher total FPKM value than in DSHS (Figure 2C,D) in total. The outlier sample ‘A-90-2’ was excluded in subsequent analyses.

### 2.3. Identification and Functional Analysis of DEGs during Different Fruit Developmental Stages

**Figure 3:**
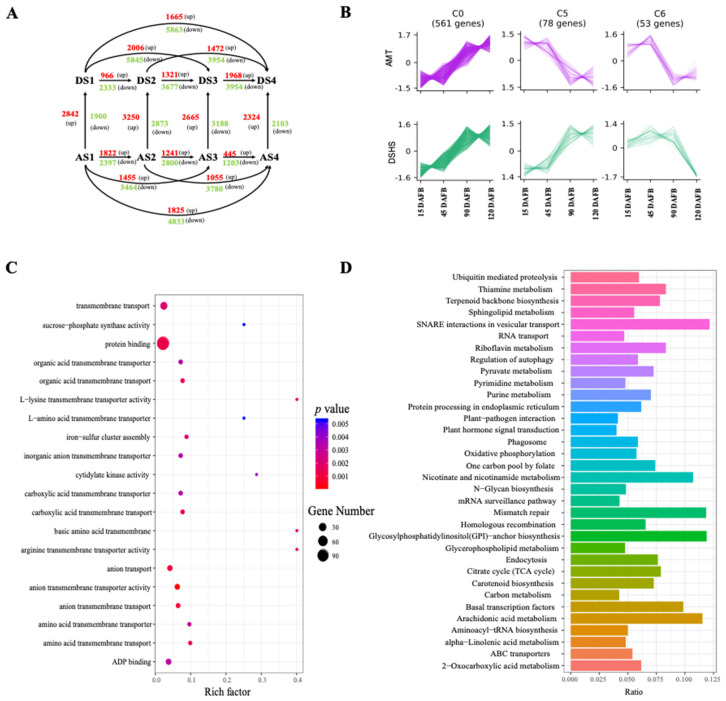
DEGs between DSHS and AMT in four fruit developmental stages and cluster analysis (**A**) Pairwise comparisons within and between two species and the developmental stages. Red numbers represent up-regulation and green number represent down-regulation. ‘A’ and ‘D’ represent AMT and DSHS, respectively. S1–S4 indicate the four stages that occur from 15 to 120 DAFB. (**B**) Cluster profiles of clusters 0, 5, and 6. The X-axis represents 15 DAFB, 45, 90, and 120 DAFB. (**C**) GO enrichment analysis of cluster 0. Bubble size indicates the number of genes, and color indicates the corrected *p*-value. The enrichment (Rich) factor represents a ratio of genes enriched in this term to the background genes belonging to this term. (**D**) KEGG enrichment analysis of cluster 0. Ratio represents number of enriched genes divided by the number of background genes number of this term.

Pairwise comparisons of gene expression levels at different stages within each variety, and gene expression levels at the same stages between the two varieties, were conducted to identify which DEGs met the criteria of FPKM > 0.1, FDR < 0.05, and |log2 fold change| >2 in each pairwise comparison. We found 18,779 DEGs that accounted for 44.39% of the total annotated genes, and the number of DEGs ranged from 455 to 5863 among comparisons (Figure 3A). In DSHS, the number of DEGs increased as the time interval between the two different stages increased. This trend was reversed in AMT. In DSHS, the number of DEGs between S1 and S2 were less than the number of DEGs between S2 and S3. When comparing two parallel stages, the number of up-regulated genes in DSHS were higher than AMT. In S1, S2 and S4, the number of up-regulated genes was higher than the number of down-regulated genes in DSHS. In these DEGs, 25 of 33 TCA-related pathway genes were differentially expressed in the parallel stage comparisons, which accounted for 76% of the total TCA-related pathway genes in pears (Table 2). Moreover, 17 TCA-related DEGs showed up-regulation in at least one stage in AMT, while 9 TCA-related DEGs showed upregulation in at least one stage in DSHS (Table 2). Of the 25 TCA-related DEGs, seven genes (*Pbr032339.1*, *Pbr033433.1*, *Pbr024294.1*, *Pbr031234.1*, *Pbr042601.1,* and *Pbr033391.1*) were shown to be constantly up-regulated in AMT during all stages. These TCA-related DEGs were predominately up-regulated in AMT, indicating that these genes may contribute to organic acid accumulation during fruit development. Additionally, many genes reported to contribute to organic acid accumulations in other plants can also be found in our DEG sets (Supplementary Table S2). For example, the *Ma1* gene is a member of the *ALMT* gene family that plays a role in transport of malic acid molecules from the cytosol to the vacuole in apple [35]. *PbrMa1* (*Pbr013272.1*) is an ortholog of apple *Ma1* (*MDP0000252114*) and may also affect the malic acid accumulation in pear. The *PbrMa1* was found to be upregulated in AMT. We also found the *PbrtDT* (*Pbr000058.1*), which is an ortholog of *AttDT* and *CsCit1*, was found up-regulated in AMT during S1, the stages of rapid organic acid accumulation.

To classify these DEGs, we performed cluster analysis in order to group the DEGs according to their expression profiles (Figure 2B, Supplementary Figure S3, Supplementary Table S3). In total, 12,267 annotated DEGs from comparisons of parallel stages between the two varieties were used for the analysis. Only 2236 genes were considered as being co-expressed in both varieties, and 21 co-expression clusters were defined (Supplementary Figure S3 and Supplementary Table S3). The same DEG clusters in two different varieties showed different expression patterns (Figure 2B, Supplementary Figure S3). For example, we can consider clusters 0, 5 and cluster 6. In cluster 0, there were 561 DEGs that exhibited the same expression pattern during S1–S4. However, in cluster 5, a total of 78 DEGs showed contrasting expression trends. In cluster 6, 53 DEGs displayed a similar trend in the early stages (S1, S2), only to show a contrasting pattern in the later stages (S3, S4). For the functional analysis, we conducted a GO enrichment analysis of all the clusters. We found the function of cluster 0 to be enriched with DEGs involved in transport, such as organic acid transmembrane transport (GO:190,3825), anion transport (GO:0006820), organic acid transmembrane transporter activity (GO:0005342), and inorganic anion transmembrane transporter activity (GO:0015103) (Figure 3C). In particular, GO:1903825, GO:0015849, and GO:0008509 are associated with organic acid transport. Transporter genes were not only enriched in cluster 0, but also in clusters 5 and 6 (Figure 3C, Supplementary Table S4). In the KEGG analysis of cluster 0 (Figure 3D), pathways related to transporters were also enriched, such as the SNARE interactions in vesicular transport and ABC transporters. Cluster 0 was also enriched in DEGs involved in the t (TCA cycle) pathway (Supplementary Table S5). Transporter genes clustered together with TCA-related pathway genes together, indicating that these transporter genes were also important for organic acid accumulation.

### 2.4. Weighted Gene Co-Expression Network Construction and Analysis

**Figure 4:**
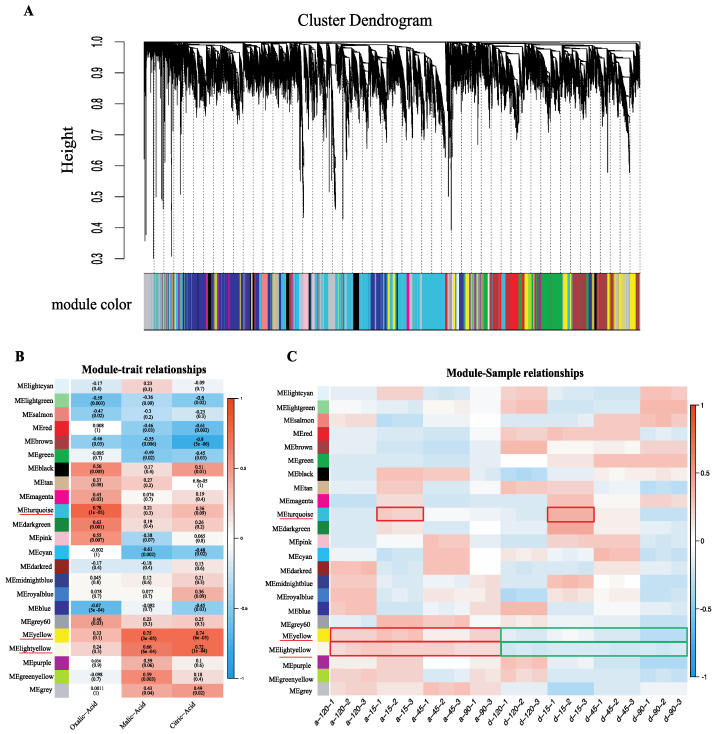
The WGCNA network analysis of 23 samples and the expression profiles of three key modules. (**A**) Dendrogram of co-expression modules identified by WGCNA. The leaves of the tree represent genes, and their annotations are represented by colors (below). (**B**) Module–acid correlation heatmap. The color scale on the right shows correlations from −1 (red) to 1 (blue). (**C**) Module–sample correlation heatmap. The color scale on the right shows correlations from −1 (red) to 1 (blue). The sample name ‘a’ represents AMT and ‘d’ represents DSHS, 15, 45, 90, 120 represents 120/15/45/90 DAFB and 1/2/3 represents three replicates.

To determine which specific genes are highly associated with fruit acidity variation between the two varieties, a weighted correlation network analysis (WGCNA) was conducted. Genes with expression values higher than 5 FPKM were used in WGCNA. The ‘a-90-2’, which was an outlier sample, was excluded from the WGCNA. In total, 27,239 expressed genes were used to construct the co-expression network. Of these, 3295 genes were not assigned to any co-expression modules, while the remaining 23,944 genes were classified into 22 distinct modules (the grey module was excluded) (Figure 4A). In module–trait correlation analysis, we found the turquoise, yellow, and light-yellow modules had the highest correlations with OA, MA, and CA, respectively (Figure 4B). The correlations between these three modules and the gene expression profiles were calculated. The gene significance (GS) scores and module membership (MM) in these modules were also highly correlated. For example, the GS and MM were closely correlated (*cor* = 0.71, *p* = 1.9 × 10^−16^) in the light-yellow module for CA (Supplementary Figure S4). GS and MM were also correlated in the yellow module (*cor* = 0.63, *p* < 1 × 10^−200^) for MA (Supplementary Figure S4). GS and MM were also correlated in the turquoise module (*cor* = 0.59, *p* < 1 × 10^−200^) for CA (Supplementary Figure S4). These results reflect the fact that not only were these three modules but the genes in these modules strongly correlated with organic acids.

**Figure 5:**
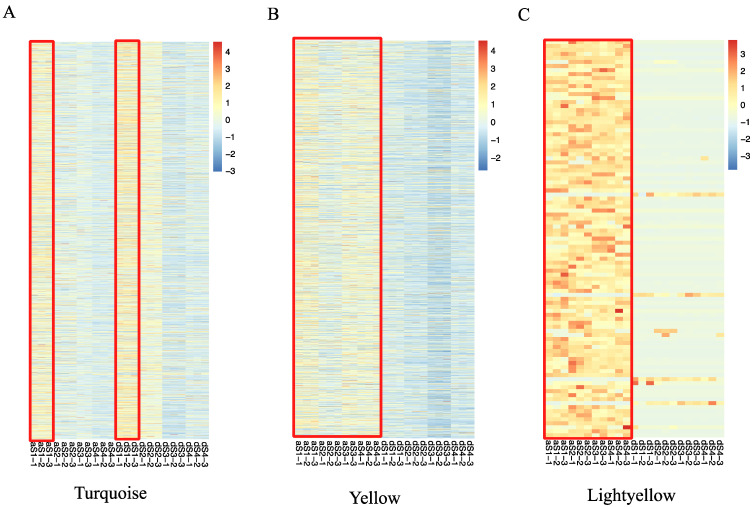
Expression heatmaps for all genes in three key modules. (**A**) The expression profile of 5988 genes in the turquoise module. Samples from aS1 and dS1 are marked by a red box. AMT is indicated by ‘a’. (**B**) The expression profile of 2191 genes in the yellow module. Samples from AMT are marked by a red box. (**C**) The expression profile of 99 genes in the light-yellow module. Samples from AMT are marked by a red box.

By examining the module–sample correlation heatmap, we also found that the turquoise, yellow, and light-yellow modules were stage-specific or sample-specific (Figure 4C). The turquoise module was specifically correlated during S1 in both DSHS and AMT. The yellow and light-yellow modules were correlated with AMT during all stages. In 23 samples, genes in the yellow and light-yellow modules were more highly expressed in AMT than in DSHS; genes in the turquoise module showed high expression in 15 DAFB in both varieties. The gene expression profiles of the three modules produced results consistent with the module–sample correlation heatmap (Figure 5A–C). Genes in the turquoise module were expressed more highly in S1 than in any other stage (Figure 5A), while in yellow and light-yellow modules, genes in AMT were expressed more highly than in DSHS. This was especially true in the light-yellow module (Figure 5B,C), which may have resulted in the higher organic acid content in AMT than in DSHS. Furthermore, nine TCA-related DEGs were found in the light-yellow (*Pbr033433.1*), turquoise (*Pbr022525.1*, *Pbr024406.1*, and *Pbr039718.1*) and yellow (*Pbr014487.1*, *Pbr022705.1*, *Pbr032250.1*, *Pbr032339.1*, and *Pbr042601.1*) modules, suggesting that these genes are highly associated with organic acid accumulation during the stages of fruit development. In terms of transporters, *PbrMa1*, which was upregulated in AMT during S2, can be found in the light-yellow module. A high correlation between MA and CA was also found in the light-yellow module, which is consistent with the function of *PbrMa1*. *Pbr005872.1*, a DEG-encoding V-type ATPase, is located in the turquoise module and highly expressed in AMT. *Pbr026279.1*, a P-type ATPase P3A subfamily, which was found to be highly expressed in AMT S3, is in the yellow module. Three key modules were therefore identified. These modules are highly associated with organic acid accumulation, and many TCA-related and transporter DEGs can be found in these modules, which serve as candidate genes for further investigation.

### 2.5. Identification of Transcriptional Regulators Networks

**Figure 6:**
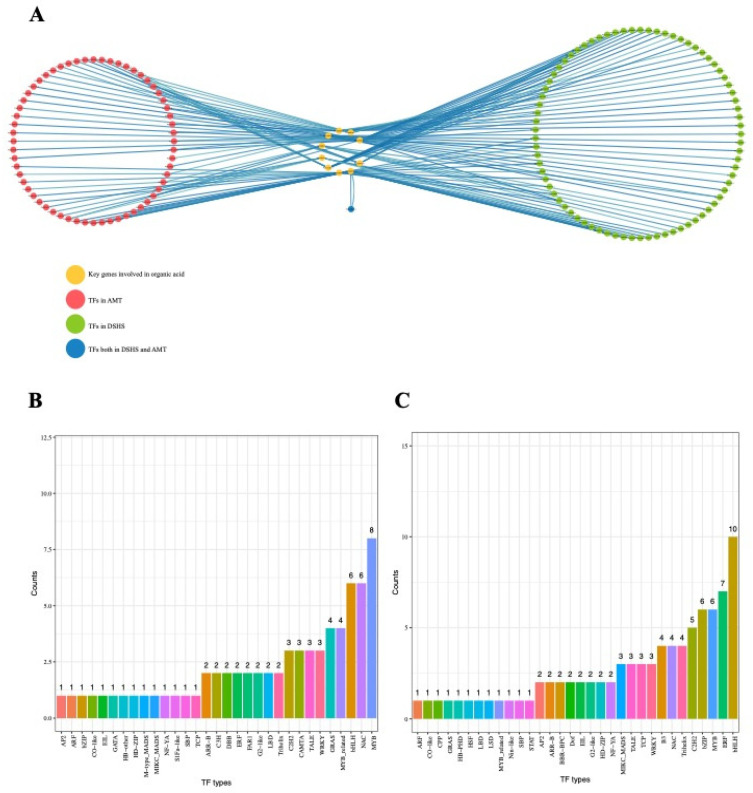
Regulatory networks between two pear varieties and statistics of TFs in two networks. (**A**) Orange circle represents candidate genes. Red and green circles represent TFs in AMT and DSHS, respectively. Blue circles represent TFs shared in both regulatory networks. (**B**) Statistics of regarding TFs types in the AMT network. (**C**) Statistics of regarding TFs types in the DSHS network.

Based on WGCNA and DEG analyses, 12 genes related to the TCA-pathway and organic acid transport were found to be differentially expressed in AMT. Located in the yellow, light-yellow, and turquoise modules, they were presumed to be key genes involved in organic acid accumulation. These genes included one *PbrMa1* (*Pbr013272.1*) gene, one *PbrVHA* gene (*Pbr005872.1*), one P-type ATPase-P3A gene (*Pbr026279.1*) and nine TCA-related genes (*Pbr033433.1*, *Pbr022525.1*, *Pbr024406.1*, *Pbr039718.1*, *Pbr014487.1*, *Pbr022705.1*, *Pbr032250.1*, *Pbr032339.1* and *Pbr042601.1*). Using these 12 genes, two transcriptional regulator networks (TRNs) were built in DSHS and AMT (Supplementary Table S7). Only one TFs (*Pbr018978.1*, TALE TFs) was shared by two TRNs, indicating that there were divergent regulatory networks between the two varieties (Figure 6A). In the DSHS network, we found a total of 86 TFs co-expressed with 12 key genes (Figure 6A). These TFs were mostly from the *bHLH* family (ten), *ERF* family (seven), *MYB* family (six), and *bZIP* family (six) (Figure 6B). In AMT network, 70 TFs were found to be co-expressed with key genes, and *MYB* (eight), *bHLH* (six) and *NAC* (six) were the top three families, containing 70 TFs (Figure 6C, Supplementary Table S8).

### 2.6. Selective Sweeps Reveal Domestication Genes in Transcriptional Regulators Networks

**Figure 7:**
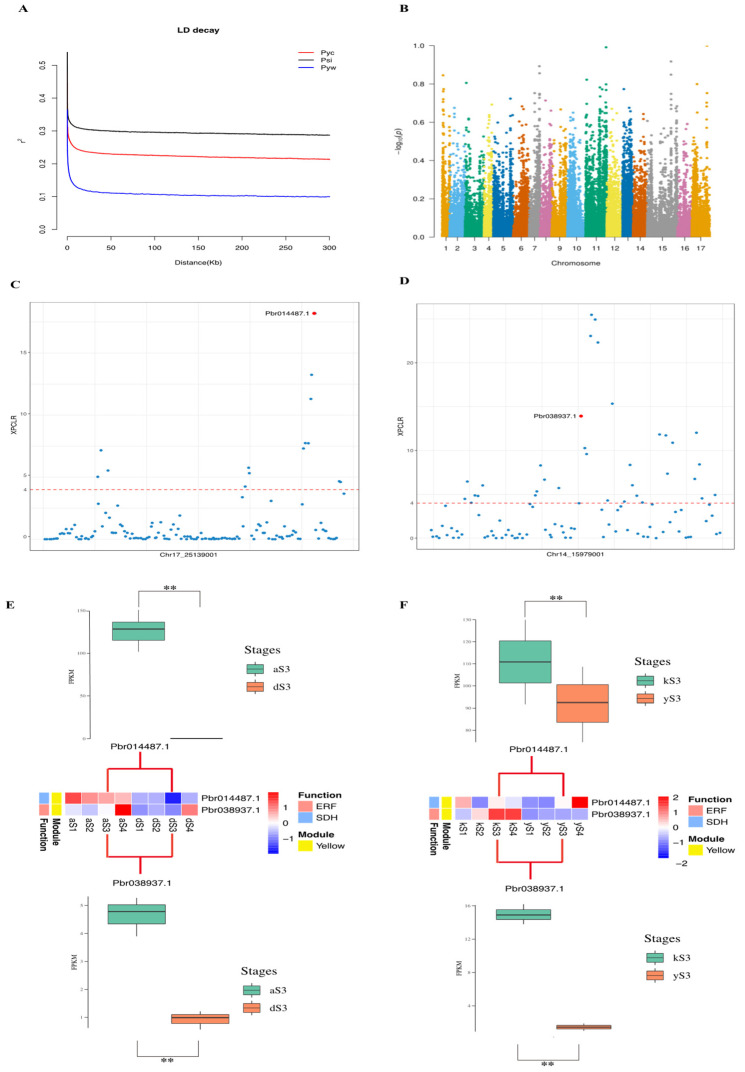
Selective signals and expression validations. (**A**) LD decay in different pear groups, including *P. bretschneideri* (red) groups, *P. sinkiangensis* (black) groups and Asian wild (blue) groups. (**B**) Distribution of XP-CLR values between *P. bretschneideri* groups and *P. sinkiangensis* groups across the whole genome of the pears. (**C**) XP-CLR signal of Pbr014487.1. (**D**) XP-CLR signal of *Pbr038937.1*. (**E,F**) Expression validation between ‘Yali’ and ‘Korla’. The upper boxplots represent the expression of *Pbr014487.1* in S3 (including three biological replicates) between two varieties of pear. The middle heatmaps are gene profiles of two genes during S1−S4. The bottom boxplots represent the expression of *Pbr038937.1* in S3 (including three biological replicates) between two varieties of pear. Wald test using DE-seq2 and Bonferroni−Holm method used to correct *p*-values, ** *FDR* < 0.01.

DSHS and AMT belong to two different domesticated pear species, *P. bretschneideri* and *P. sinkiangensis*, respectively [31]. The contrasting phenotypes between these two varieties such as fruit flavor, fruit shape, hinted that they have undergone differential selection [31]. We examined the selective sweeps between *P. bretschneideri* and *P. sinkiangensis* groups using previous data (NCBI accession number: PRJNA381668) [31]. The LD decay for two Asian pear subgroups (*P. bretschneideri* and *P. sinkiangensis*) was much slower than in the wild, indicating that both Asian subgroups had undergone selection during domestication (Figure 7A). Therefore, we further identified genes in two TRNs that overlapped with selective sweep regions. We found 15,570 and 5003 candidate selective sweeps can be found in the *F_st_* and XP-CLR analyses, respectively (Figure 7B, Supplementary Figure S5). Two genes were found located in both selective sweep regions (Figure 7C,D): *Pbr014487.1*, a TCA-related gene encoding OGDH, and ERF TF (*Pbr038937.1*). Both genes were upregulated in AMT during S3. The expression of these genes were altered during the domestication process [28,36]. In maize, the expression of the *tb1*, *gt1*, *tru1*, and *ra1* genes were changed during domestication, which is consistent with our results [37,38,39,40].

To confirm this assumption, the expression levels of these genes were investigated in other two pear cultivars, *P. sinkiangensis* cv. ‘Korla’ and *P. bretschneideri* cv. ‘Yali’ (Figure 7E,F). In S3, two genes were up-regulated in ‘Korla’, which validates the previous results. *P. bretschneideri* group and *P. sinkiangensis* may have experienced different selection processes due to the different geographic regions from which they originate. However, the low expressions in DSHS and ‘Yali’ indicates these two genes did experience selection effects as a result of the important roles they play in controlling pear acidity.

**Figure 8:**
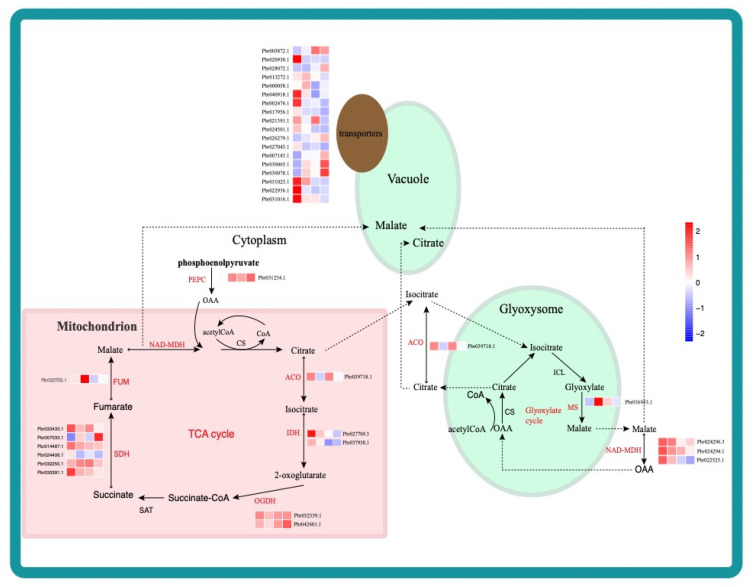
Expression profiles of DEGs related to the TCA and glyoxysome pathways. Schematic illustrations of TCA and glyoxysome pathways. DEG names and expression levels in AMT are presented in the pathway. The sky-blue frame represents the cell membrane, pink represents a mitochondrion, and the green circle represents the glyoxysome. The color scale from red to blue corresponds to expression levels from high to low, respectively, based on the log2(FPKM). SDH: succinate dehydrogenase, ACO: aconitase; FUM: fumarase; IDH: isocitrate dehydrogenase; MS: malate synthase; ICL: isocitrate lyase; NAD-MDH: NAD-Malate dehydrogenase; PEPC: phosphoenolpyruvate carboxylase; OGDH: ketoglutarate dehydrogenase; CS: citrate synthetase. Names of genes identified from WGCNA are marked in red.

A total of 36 DEGs, including 12 TCA-related genes and 18 transporters, were identified via the DEG analysis (Figure 8). Eight of the twelve TCA-related genes and three of the transporters were selected as candidate genes because they were located in three key modules (Figure 8, marked as red names). By introducing selective sweeps, two domestication genes (*Pbr014487.1*, *Pbr038937.1*) were identified. Although the two domestication genes need to be focused, these TCA-related and transport DEGs identified from WGCNA are also important. The TFs in TRNs can also affect organic acid acculation via protein–protein interactions with these DEGs.

### 2.7. qRT-PCR Validation

**Figure 9:**
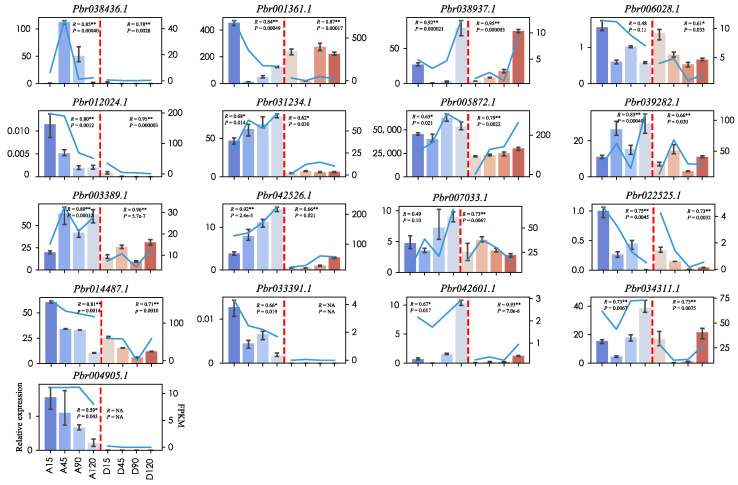
Validation of expression patterns by qRT-PCR of selected DEGs from RNA-seq analysis. The left Y-axis represents relative expression levels and their corresponding bar plot with standard deviations (denoted as an error bar in each plot). The right Y-axis represents expression levels calculated by fragments per kilobase per million reads (FPKM) method corresponds line plot in each plot. Additionally, the X-axis in each plot means successive developmental stages in two pear varieties. A/D15: 15 DAFB in ‘Amute’/ ‘Dangshansuli’ pear. A/D45: 45 DAFB in ‘Amute’/ ‘Dangshansuli’. A/D90: 90 DAFB in ‘Amute’/ ‘Dangshansuli’. A/D120: 120 DAFB in ‘Amute’/ ‘Dangshansuli’. R means Pearson Correlation Coefficient (PCC) between FPKM and relative expression, and P means *p*-value of PCC, * *p* < 0.05; ** *p* < 0.01.

To validate their status as DEGs as determined from the RNA-seq data, a total of 17 DEGs were selected for further analysis by qRT-PCR, and specific primers were designed for these genes (Supplementary Table S9). To estimate the correlation between FPKM and the relative expression values, the PCCs were calculated. The highest PCC value was 0.96 (*Pbr003389.1*), and at least nine genes showed PCC values higher than 0.8. The relative expression trend for most of the genes were highly consistent with the RNA-seq data (Figure 9). These results confirmed that our transcriptome data could serve as a foundation for further downstream analyses. The relative expression of these two domestication genes was also consistent with RNA-seq data.

## 3. Discussion

### 3.1. Divergent Gene Expression Contributes to Phenotypic Differences

The organic acid content (sugar/acid ratio) plays an important role in determining fruit flavor and palatability. In this study, we characterized the changes in three major types of organic acids during successive fruit developmental stages in high- and low-acidity pear varieties by employing UHPLC. We detected significant differences in the organic acid content between the two pear varieties. The content of MA and CA, which are two predominant organic acids in pear fruit [32], was higher than in AMT than in DSHS at different development stages. Previous studies have shown that alterations in gene expression play crucial roles in driving phenotype changes during the evolution and domestication of different plant species [29,41,42,43]. In soybean, the differential expression of a large number of genes at 3–5 days after fertilization results in differences in soybean seed size [43]. In maize, the *tb1* gene was enhanced in the cultivated variety (*Zea mays* ssp. mays), leading to the repression of axillary growth and increased apical dominance [44]. Changes in the expression of the *ZmCCT10* gene can also affect the flowering time of maize [45]. In tomato, phenotypical differences between the wild and cultivated varieties correlate with gene expression profiles [29]. For example, the significant wax differences between wild and cultivated tomatoes may be due to higher expression among the genes that involved in epicuticular waxes, including ECERIFERUM 1 (*CER1)*, ECERIFERUM 2(*CER2*), ECERIFERUM 6 (*CER6*) and ECERIFERUM 10 (*CER10*) in wild varieties compared to their cultivated counterparts [29].

Comparative transcriptome analysis is a useful approach for discovering important clues about gene function and the molecular basis of developmental processes [46]. In this study, by performing WGCNA, not only were three modules identified that were closely related with the content of three organic acids, but these modules were also found to be closely associated with AMT samples and specific developmental stages.

### 3.2. Divergent Gene Expression Influenced by Differential Selection Process

Throughout history, the basic principle behind the efficient domestication and improvement of crops has been to select plants based upon their phenotypes. [47]. People will select for preferred traits in their crops. For example, European pear and Asian pear have distinct phenotypic traits, including fruit acidity, shape, sugar, and stone cells, among others [31]. One model proposes that many trait-related genes were under selections, which caused the differences between two pear groups. Here, we used resequencing data to examine the selective sweep regions between two populations, *P. sinkiangensis* and *P. bretschneideri*. Two domestication genes were found by integrating WGCNA and selective sweeps. Differences in expression between the two varieties were determined by investigating the expression levels in AMT and DSHS, as well as in ‘Yali’ (*P. bretschneideri*) and ‘Korla’ (*P. sinkiangensis*). *Pbr014487.1* was a gene encoding SDH which is a gene related to TCA pathway. Genes in the TCA pathway have already been reported to control fruit acidity [4,5,48,49,50]. The *SlSDH2-2* gene in tomatoes was reported to affect organic acid levels [51]. In apple, overexpressing *MdcyMDH* can also result in malate accumulation [4]. PEPC in apple positively regulate malic acid accumulation in apple [5]. *Pbr038937.1* was an ERF TFs. Many TFs were already identified can control fruit organic acidity. In citrus, *CitERF13*, *CitNAC62*, *CitWRKY1*, and *CrMYB73* were able to control fruit acidity [52,53,54]. In apple, *MdMYB44* and *MdMYB73* can affect fruit malate content [34].

Future selective sweeps will presumably identify additional candidate genes. However, only two domestication genes were found. This may be due, in part, to the low coverage of resequencing data. Another may because the relatively weak selection during pear domestication [31]. We need emphasis that these DEGs located in three key modules related to the TCA pathway and transport should be treated as candidate genes for the regulation of fruit acidity. The gene-*TF* networks also play important roles in regulating these genes.

## 4. Materials and Methods

### 4.1. Plant Sample Collection

Two different pear varieties with similar product maturity period, ‘Dangshansuli’ (DSHS, *Pyrus bretschneideri* cv. Dangshansuli) and ‘Amute’ (AMT, *Pyrus sinkiangensis* cv. Amute) (AMT), were selected for this study. ‘Dangshansuli’ and ‘Amute’ were collected from Gaoyou Farm in Yangzhou, Nanjing, China and the Institution of Pomology of The Chinese Academy of Agricultural Sciences (CAAS) in Xingcheng, Liaoning, China, respectively. Fruit from both varieties were collected at 15 days after full blooming (15 DAFB, S1), 45 DAFB (S2), 90 DAFB (S3), and 120 DAFB (S4). The peel of the fruit was removed, and the flesh was quickly cut into liquid nitrogen and stored in an ultra-low freezer (−80 °C).

### 4.2. Determination of Organic Acid Contents

MA, CA, and OA were extracted and measured following the reported method [13]. Then, the samples were analyzed using ultra high-performance liquid chromatography (UHPLC, Thermo Ultimate 3000; MA, USA). A 25-mM phosphate buffer (pH 2.4) was used as the mobile phase at a flow rate of 0.5 mL min^−1^ and 30 °C. The three acids’ concentrations in each sample by comparing the area of peak with the known standard concentration.

### 4.3. RNA Isolation, Library Construction and Sequencing

Total RNA from two varieties developmental stages were extracted using the RNAprep Pure Plant Kit (Polysaccharides & Polyphenolics-rich) (Tiangen, Beijing, China). All sample were replicated three times. Twenty-four libraries were constructed using NEBNext UltraTM RNA Library Prep Kit for Illumina (NEB, MA, USA) following manufacturer’s recommendations. Sequencing on an Illumina platform and paired-end reads were generated.

### 4.4. RNA-seq Data Processing

First, adapter sequences, poly (A/T) tails, and reads with quality scores lower than 15 were removed using fastp (v0.20.0-6ff0ffa) software [55]. Then, clean reads were mapped to the reference genome using Hisat2 [56] with the following parameters: -I 0; -X 500; −phred33; –min-intronlen 20; –max-intronlen 500,000. Mapped reads were counted using FeatureCounts for each sample [57]. Gene expression levels were estimated the FPKM values using our in-house python script by following the formula: FPKM = total exon fragments/mapped reads (Millions)×exon length (KB). DEGs were identified using DEseq2 [58]. Gene clustering analysis performed by Clust [59]. GO and KEGG enrichment using TopGO and ClusterProfiler [60,61].

### 4.5. RNA-seq Data De Novo Assembly and Phylogenetic Analysis

Clean reads from ‘Bartlett’ (*Pyrus communis* cv. Bartlett), DSHS and AMT were de novo assembled using Trinity v.2.8.5 with default parameters. Briefly, reads in each sample will be normalized to remove excess reads beyond 200 coverage. Jellyfish was employed to generate *k*-mer catalogs from reads. Inchworm was used to construct linear contig from *k*-mers. Chrysalis can cluster contigs and generate de Bruijn graph. Finally, assembling clusters of reads involved in inchworm, chrysalis and butterfly which incorporate in Trinity packages.

Then assembled transcriptome data were first remove redundant contigs using cd-hit v.4.8.1 [62]. We chose 95% sequence identity as the value to use for this study. Further reducing assembly mistakes and redundant by comparison to protein sequences from five sequenced and annotated plant genomes from GDR (peach, strawberry, apple, European pear, and Chinese white pear). Using diamond blastx [63], the transcripts did not have any best hit protein were removed. Then, Transrate v.1.0.3 36 was used to find the longest open reading frame (ORF) and translate genes to protein sequences.

Then we identified single-copy genes to perform phylogenetic analysis. Orthofinder v.2.1.2 37 was used to found single-copy genes among assembled ‘Bartlett’ (*Pyrus communis*), DSHS and AMT. Genomes of *Arabidopsis thaliana*, *Fragaria verca*, *Pruns persica,* and *Malus domestica* were downloaded from GDR (https://www.rosaceae.org/, accessed on 14 July 2020) as outgroups. RAxML v8.2.12 [64] was employed to build maximum likelihood (ML) concatenation species tree and ASTRAL-II v.5.7.1 [65] was used to estimate the species tree on the basis of all single-copy genes.

### 4.6. Resequencing Data Processing and Detecting Selective Signals

Resequencing data of 113 pear accessions was obtained from NCBI database (https://www.ncbi.nlm.nih.gov/bioproject, accessed on 13 April 2019) under accession PRJNA381668 [31]. The SNP calling step was essentially followed the Genome Analysis Toolkit (GATK) Best Practices [66]. The high quality paired-end reads were mapped to the Asian pear genome ‘Dangshansuli’ using BWA-MEM [67]. PCR duplicates were marked by MarkDuplicates implemented in Picard tools (http://broadinstitute.github.io/picard/, accessed on 22 August 2019). We performed SNP calling step using HaplotypeCaller which implemented in GATK4 (Genome Analysis Toolkit, 4.1.1.0-ea3032d) [68]. Raw variant data were separated to SNPs and indels using SelectVariants in GATK. GATK Hard-filtering was performed with the following parameters: MappingQualityRankSum of <−12.5, polymorphism confidence scores (QUAL) <30, genotype call quality divided by depth (QD) < 2.0, Phred-scaled *P* value of Fisher exact test for strand (FS) > 60.0, mapping quality (MQ) < 40.0, and ReadPositionRankSum of <−8.0. VCFtools was used to perform a further filter to generate high-quality SNPs.

Fixation index (*F_ST_*) was calculated using VCFtools with a 10 kb sliding windows with step size 1 kb. We used the cross-population composite likelihood ratio (XP-CLR) score to confirm the selective sweeps on the basis of domestication features with window size 10 kb and step size 1 kb.The windows with (1) the top 1% of XP-CLR values and (2) the top 5% of *F_st_* values were considered to be our candidate selective sweep regions. Genes which located in candidate selective sweep regions were candidate selected genes.

### 4.7. Weighted Correlation Network Analysis (WGCNA) and Co-Expression Network Construction

A weighted gene co-expression network was constructed using R package WGCNA [69]. Genes that were expressed in both varieties were selected in this analysis. In total, 27,239 genes with suitable FPKM values were used to construct an unsigned gene co-expression network. The soft threshold in this study was 14, the TOMtype was unsigned, the minModuleSize was 40, and the mergeCutHeight was 0.3.

Mutual rank (MR) score which was calculated based on Pearson’s correlation coefficient (PCC) was used to build co-expression network between TFs and genes [70]. The mutate rank was calculated as the geometric mean of the rank of the PCC of gene A to gene B and of the gene B to gene A. MR scores treated as network edge weights. Any edge with PCC less than 0.3 and single direction rank of PCC higher than 5 were excluded in co-expression networks.

### 4.8. qRT-PCR Validation

To validate the RNA-seq data, quantitative reverse transcription-PCR (qRT-PCR) was employed. Total RNA from 24 samples (2 varieties, 4 stages with 3 replicates) were extracted and reverse-transcribed to cDNA. The specific primers for the genes were designed using Primer Premier 5.0 software (Premier Biosoft Interpairs, Palo Alto, CA, USA). Pyrus Tube1 was selected as the internal reference gene. All the samples were analyzed on a LightCycler 480 instrument (Roche, BSL, Switzerland), and expression levels were calculated using the 2^−ΔΔCt^ method [71].

## 5. Conclusions

Using DEG analysis and gene clustering, the TCA-related pathway and transporter genes were determined to play important roles in organic acid accumulations. By performing WGCNA and selective sweep analyses, domestication genes, and their potential regulatory networks were identified. These results revealed that domestication may have led to organic acid variation in pears by changing their gene expression levels and provided candidate genes which related to organic acid accumulation that could be targeted for manipulating fruit acidity. Additionally, selections were widely existing [28], meaning that the genes that control other important traits, such as stone cells, sugar content, in pear could be uncovered by employing similar methods.

## Figures and Tables

**Table 1:** Digital descriptions of organic acid contents. Digital descriptions of organic acids content in Figure 1 (average ± SD).

Organic Acid Type	Varieties	S1	S2	S3	S4
OA	AMT	1.3182 ± 0.22	0.7615 ± 0.44	0.6233 ± 0.11	0.1669 ± 0.0085
	DSHS	0.8982 ± 0.12	0.8406 ± 0.040	0.2305 ± 0.048	0.1959 ± 0.0084
MA	AMT	6.543 ± 0.35	2.195 ± 0.64	6.232 ± 0.39	4.310 ± 0.50
	DSHS	3.045 ± 0.11	2.153 ± 0.16	2.920 ± 0.034	2.178 ± 0.093
CA	AMT	1.927 ± 0.15	1.443 ± 0.15	1.606 ± 0.19	1.574 ± 0.082
	DSHS	1.412 ± 0.16	1.116 ± 0.11	1.265 ± 0.12	0.7402 ± 0.068

**Table 2:** Details of TCA-related DEGs ‘A’ and ‘D’ represent AMT and DSHS, respectively. S1–S4 indicate the four stage that occur from 15 to 120 DAFB. (+) means gene up-regulated in AMT and (−) means gene down-regulated in AMT.

Genes	Function	DS1 vs. AS1	DS2 vs. AS2	DS3 vs. AS3	DS4 vs. AS4
Pbr039718.1	ACO			+	
Pbr008056.1	ACO			−	
Pbr022705.1	FUM	+		+	+
Pbr037938.1	IDH			-	+
Pbr027709.3	IDH			+	
Pbr024296.3	MDH	+		+	+
Pbr024294.1	MDH	+	+	+	+
Pbr002553.1	MDH				−
Pbr024269.1	MDH			−	
Pbr022525.1	MDH			+	
Pbr011434.1	MDH	−			
Pbr027217.2	MS	−	−		
Pbr036933.1	MS		+		
Pbr032339.1	OGDH	+	+	+	+
Pbr042601.1	OGDH	+	+	+	
Pbr015662.1	OGDH	−	−	−	−
Pbr031234.1	PEPC	+	+	+	+
Pbr017951.1	SDH			−	
Pbr033433.1	SDH	+	+	+	+
Pbr022657.1	SDH	−			
Pbr007033.1	SDH				+
Pbr014487.1	SDH			+	
Pbr024406.1	SDH		−	+	
Pbr032250.1	SDH		+		
Pbr033391.1	SDH	+	+	+	+

## Data Availability

All genome sequences and resequencing data can be found in NCBI Bioproject (PRJNA157875 and PRJNA381668) and genome sequences also available at Pear genome project (http://peargenome.njau.edu.cn/).

## Supplementary Materials

Supplementary materials can be found at https://www.mdpi.com/article/10.3390/ijms22179622/s1.
